# Extracellular cathepsin Z signals through the α_5_ integrin and augments NLRP3 inflammasome activation

**DOI:** 10.1016/j.jbc.2021.101459

**Published:** 2021-12-02

**Authors:** Rhiannon I. Campden, Amy L. Warren, Catherine J. Greene, Jose A. Chiriboga, Corey R. Arnold, Devin Aggarwal, Neil McKenna, Christina F. Sandall, Justin A. MacDonald, Robin M. Yates

**Affiliations:** 1 Department of Biochemistry and Molecular Biology, Faculty of Medicine, University of Calgary, Calgary, Alberta, Canada; 2 Snyder Institute for Chronic Disease, University of Calgary, Calgary, Alberta, Canada; 3 Department of Veterinary Clinical and Diagnostic Sciences, Faculty of Veterinary Medicine, University of Calgary, Calgary, Alberta, Canada; 4 Department of Comparative Biology and Experimental Medicine, Faculty of Veterinary Medicine, University of Calgary, Calgary, Alberta, Canada

**Keywords:** arginine-glycine-aspartic acid (RGD) domain, cathepsin Z, inflammasome, inflammation, integrin, interleukin-1 (IL-1), NLRP3, silica, silicosis, APC, antigen-presenting cells, BMDC, bone marrow-derived dendritic cells, EAE, encephalomyelitis, FBS, fetal bovine serum, LPS, lipopolysaccharide, MS, multiple sclerosis, MSU, monosodium urate, PMA, phorbol myristate acetate, RGD, arginine-glycine-asparigine

## Abstract

Respiratory silicosis is a preventable occupational disease that develops secondary to the aspiration of crystalline silicon dioxide (silica) into the lungs, activation of the NLRP3 inflammasome, and IL-1β production. Cathepsin Z has been associated with the development of inflammation and IL-1β production; however, the mechanism of how cathepsin Z leads to IL-1β production is unknown. Here, the requirement for cathepsin Z in silicosis was determined using WT mice and mice deficient in cathepsin Z. The activation of the NLRP3 inflammasome in macrophages was studied using WT and cathepsin Z-deficient bone marrow-derived murine dendritic cells and the human monocytic cell line THP-1. The cells were activated with silica, and IL-1β release was determined using enzyme-linked immunosorbent assay or IL-1β bioassays. The relative contribution of the active domain or integrin-binding domain of cathepsin Z was studied using recombinant cathepsin Z constructs and the α_5_ integrin neutralizing antibody. We report that the lysosomal cysteine protease cathepsin Z potentiates the development of inflammation associated with respiratory silicosis by augmenting NLRP3 inflammasome-derived IL-1β expression in response to silica. The secreted cathepsin Z functions nonproteolytically *via* the internal integrin-binding domain to impact caspase-1 activation and the production of active IL-1β through integrin α_5_ without affecting the transcription levels of NLRP3 inflammasome components. This work reveals a regulatory pathway for the NLRP3 inflammasome that occurs in an outside-in fashion and provides a link between extracellular cathepsin Z and inflammation. Furthermore, it reveals a level of NLRP3 inflammasome regulation that has previously only been found downstream of extracellular pathogens.

Respiratory silicosis (silicosis) is an inflammatory disease initiated by inhalation and deposition of crystalline silica into the lungs. The deposited silica leads to the activation of alveolar macrophages, alveolar proteinosis, and fibrosis, which compromises lung function (1). Silicosis can be either chronic or acute, where chronic cases tend to occur because of small volumes of silica dust exposure over a long period (2). Acute cases typically result from significant volume exposures (2). Two million workers in the USA are at risk for developing silicosis due to occupational hazards, including mining, pottery making, road construction, and stone masonry, although the incidence of silicosis is relatively low, with an average of 0.58 cases per million within the total population (3).

The primary driver of silicosis is thought to be the alveolar macrophage, which responds to phagocytosed silica crystals by releasing inflammatory mediators (1). In particular, the release of IL-1β through NLRP3 inflammasome activation has been shown to enhance the inflammatory microenvironment surrounding the deposited silica crystals, driving pulmonary inflammation and the associated pathology (2, 4, 5, 6, 7). In the context of neuroinflammation, we have previously shown that the lysosomal cysteine protease cathepsin Z potentiates IL-1β release by macrophages and that deletion of cathepsin Z is protective in the mouse model of multiple sclerosis (MS) and experimental autoimmune encephalomyelitis (EAE) (8). This finding is particularly relevant because epigenetic upregulation of cathepsin Z in humans has been proposed as a risk factor for MS (9). Although the pathophysiologies of MS and EAE are multifactorial and complex, we set to explore the role of cathepsin Z on the NLRP3 inflammasome in the context of silicosis, starting with the hypothesis that cathepsin Z amplifies inflammation and pathology in silicosis through the enhancement of NLRP3-dependent generation of IL-1β.

Cathepsin Z is unique among the lysosomal cysteine cathepsin family, as it is a carboxyexopeptidase with strict monopeptidase activity and contains a short prodomain that is covalently bound into the active-site cysteine by a disulfide bond (10, 11, 12). To be activated as a protease, the active site must be reduced and the prodomain cleaved by another protease such as cathepsin L (12). Cathepsin Z also contains an integrin-binding domain (Arg-Gly-Asp; RGD) within its prodomain (13). Thus, cathepsin Z may function through its capacity to bind integrins or to act as an active exopeptidase. Although cathepsin Z has been implicated in NLRP3-inflammasome activation, the mechanism through which this is achieved had yet to be determined (8, 14).

The NLRP3 inflammasome requires two signals for activation: signal 1 (or priming), which can be accomplished by activation of the TLR4 receptor by lipopolysaccharide (LPS) among others and signal 2 (or activation), where a diverse array of stimuli including ATP, pore-forming toxins and crystals, such as monosodium urate (MSU) and silica, lead to NLRP3 oligomerization, caspase-1 activation, and IL-1β maturation (4, 15, 16). The mechanisms by which lysosomal cathepsins intersect with NLRP3 inflammasome activation pathways remain contentious (17). Early evidence suggested that cathepsin B was released from the lysosome and led to activation of the NLRP3 inflammasome downstream of the initial NLRP3 stimulus; however, cathepsins are not involved in NLRP3 inflammasome activation with all NLRP3-activating stimuli (14, 18, 19, 20). It also appears that multiple cathepsins can compensate for each other during NLRP3 inflammasome activation (14). Cathepsin Z, however, appears to play a unique role in inflammasome activation that cannot be compensated for by other cathepsins (8, 14). Antigen presenting cells (APCs) that lack cathepsin Z secrete lower levels of IL-1β in response to the NLRP3-activating stimuli ATP and MSU but have equivalent levels of IL-1β mRNA to WT (8). This suggests a unique function for cathepsin Z in IL-1β generation by the NLRP3 inflammasome.

Herein, we investigated the specific contribution of cathepsin Z to IL-1β mediated inflammation in chronic respiratory silicosis. Cathepsin Z-deficient mice exhibited reduced levels of chronic inflammation within lung tissue after aspiration of crystalline silica compared with WT animals. We found that cathepsin Z was secreted by activated macrophages and that extracellular cathepsin Z acted through the α_5_ integrin to enhance the generation of IL-1β after NLRP3 inflammasome activation with silica. Together, these data also describe an immunologic mechanism for the nonredundant involvement of cathepsin Z in NLRP3-mediated inflammasome activation and adds to the growing body of evidence that cathepsins can function in the extracellular environment as modulators of inflammation.

## Results

### Cathepsin Z promotes inflammation in a mouse model of silicosis

**Figure 1:**
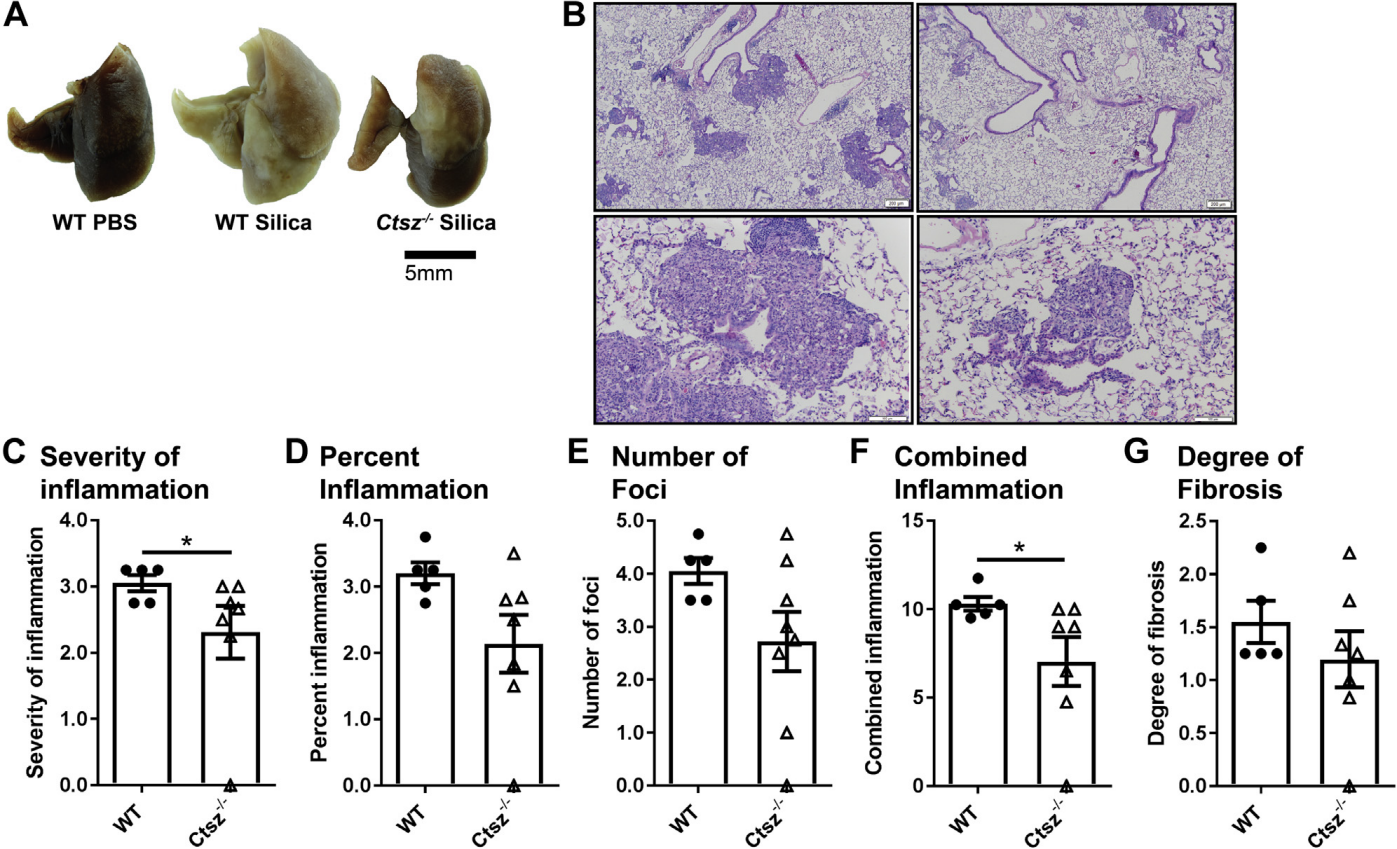
**Cathepsin Z is required for the development of inflammation in a mouse model of silicosis.** The samples were taken 90 days post oropharyngeal aspiration of 10 mg silica or vehicle control (saline). *A*, representative images of WT and Ctsz^−/−^ lungs. The scale bar represents 200 μm (*top panels*) and 100 μM (*bottom panels*). *B*, images of H&E-stained WT (*left*) and Ctsz^−/−^ (*right*) silicotic lungs. *C*–*G*, histological scoring of mouse lungs made relative to the % silica per lung and WT for each experiment (n = 5–7). The data are presented as means ± SEM ∗*p* < 0.05 *versus* WT controls (Mann–Whitney U test).

We have previously demonstrated that mice deficient in cathepsin Z exhibit lower degrees of neuroinflammation in a mouse model of MS, EAE, and generate lower levels of IL-1β after exposure of APCs to the NLRP3-activating stimuli ATP and MSU (8). Because NLRP3-generation of IL-1β has been implicated in the development of respiratory silicosis, we set to determine whether the absence of cathepsin Z is protective in a mouse model of chronic respiratory silicosis (7). Ninety days after the single aspiration of silica crystals into the proximal portions of the mouse lungs, cathepsin Z deficient (*Ctsz*^−/−^) mice showed lower levels of inflammation compared with WT mice. This was reflected by both a reduction in the gross pathology (Fig. 1*A*) and a reduction in microscopic pulmonary inflammation (Fig. 1*B*). There was a significant reduction in the histopathology scores for the severity of inflammation and the combined inflammation score in *Ctsz*^−/−^ mice compared with WT animals (Fig. 1, *C* and *F*). We also observed lower (but not significantly different) levels of percent inflammation, number of foci, and fibrosis (Fig. 1, *D*, *E* and *G*). There was no difference between IL-1β and caspase-1 mRNA levels between WT and *Ctsz*^−/−^ animals (Fig. S1, *A* and *B*). This corresponds with a phenotype we have observed in bone marrow-derived macrophages, where IL-1β protein released from *Ctsz*^*−/−*^ bone marrow-derived macrophages was decreased, but mRNA levels were not significantly different between WT and *Ctsz*^−/−^ mice (8). Cathepsin Z mRNA was increased in lungs from WT animals treated with silica compared with untreated animals, suggesting a role for cathepsin Z in silica-activated inflammation (Fig. S1*C*). Furthermore, because silicosis is mediated in part by the NLRP3 inflammasome (4), these findings are consistent with the involvement of cathepsin Z in the activation of the NLRP3 inflammasome.

### Cathepsin Z enhances the generation of IL-1β following NLRP3 inflammasome activation with silica

**Figure 2:**
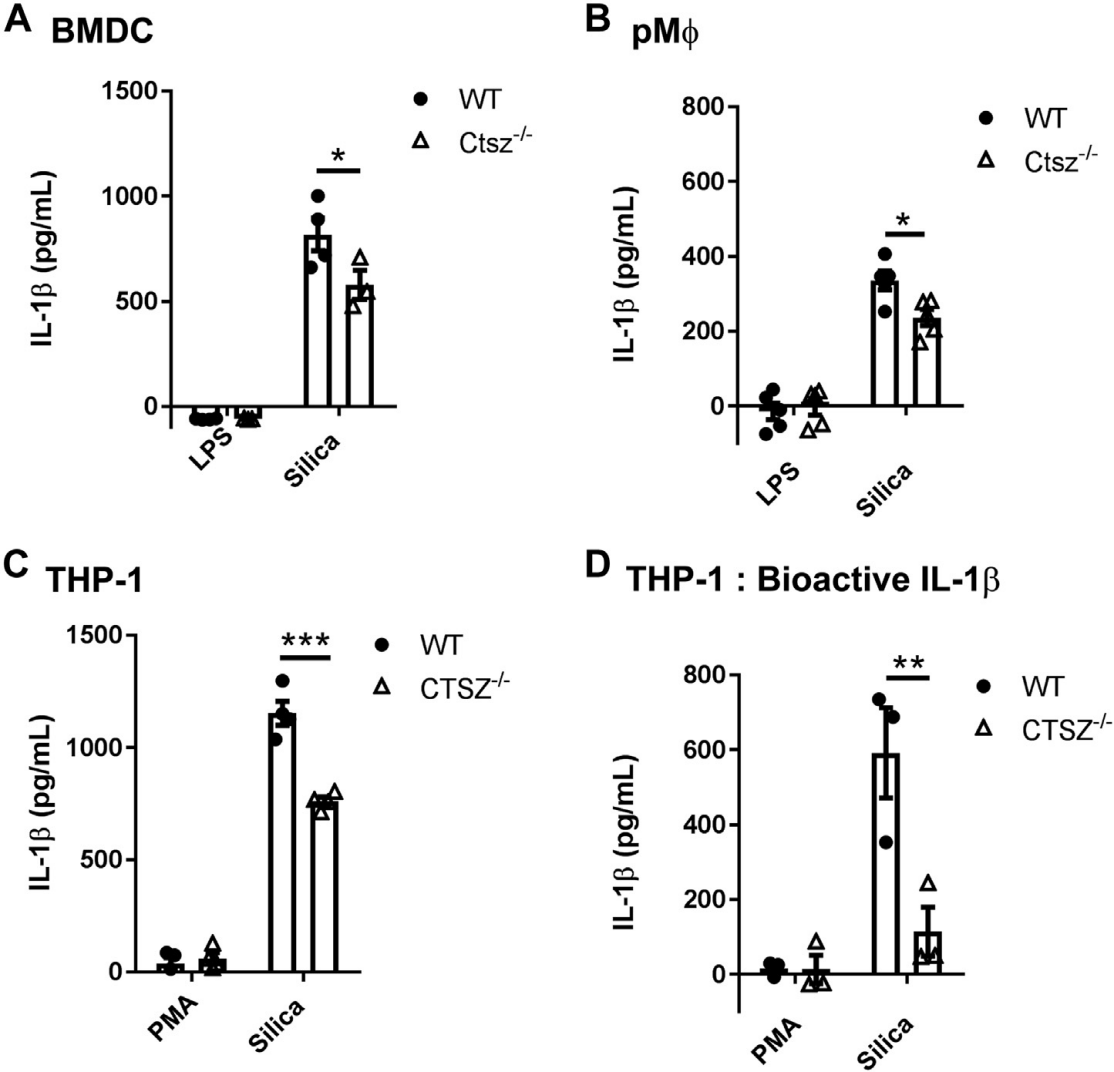
**Cathepsin Z impacts the generation of IL-1β from antigen presenting cells.***A*–*C*, IL-1β released from WT and Ctsz^−/−^ (*A*) BMDC, (*B*) pMØ, and (*C* and *D*) THP-1 cells after activation with 10 nM PMA followed by 128 μg of silica for 6 h. *A*–*C*, IL-1β measured by ELISA (n = 3–5). *D*, bioactive IL-1β released from WT and CTSZ^−/−^ THP-1 cells measured by IL-1R expressing HEK-Blue reporter cells HEK-Blue (n = 5). The data are presented as means ± SEM ∗*p* < 0.05, ∗∗*p* < 0.01, ∗∗∗*p* <0.001 *versus* WT controls (two-way ANOVA followed by Bonferroni corrected two-tailed Student’s *t* test). BMDC, bone marrow-derived dendritic cells; LPS, lipopolysaccharide; PMA, phorbol myristate acetate; pMØ, peritoneal macrophages.

**Figure 3:**
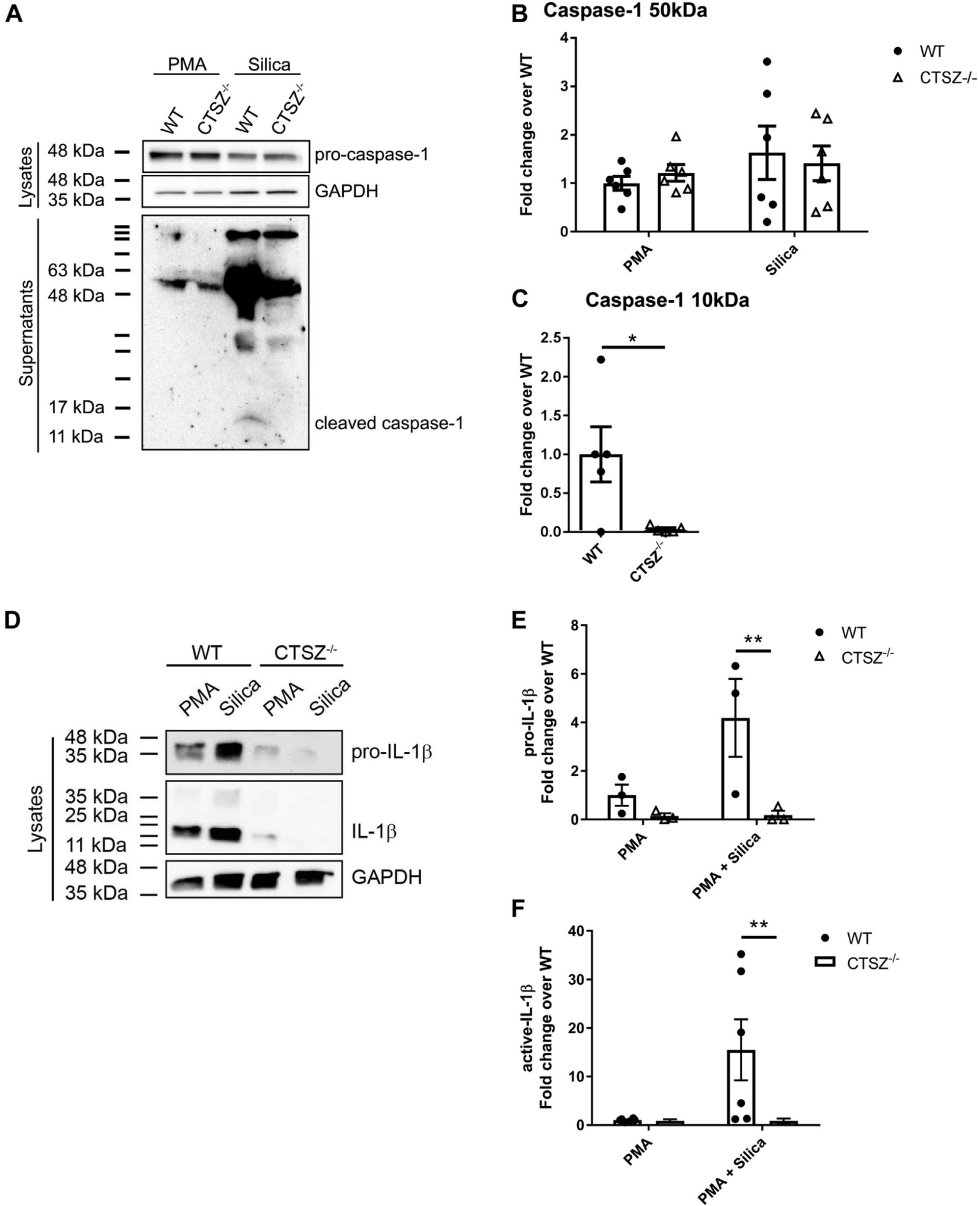
**Knockout of cathepsin Z in THP-1 cells leads to reduced active IL-1β and caspase-1 released into the supernatant after NLRP3 inflammasome activation.***A* and *D*, representative Western blot of caspase-1 (*A*) and IL-1β (*D*) from the lysates and supernatants of WT and CTSZ^−/−^ THP-1 cells treated with 10 nM phorbol myristate acetate (PMA) for 16 h after 128 μg of silica for 6 h. *B*, *C*, *E*, and *F*, quantification using densitometric analysis of total protein blots of (*B*) pro-caspase-1, (*C*) active caspase-1 (n = 5–6), (*E*) pro-Il-1β, and (*F*) active IL-1β (n = 3–5). The data are presented as means ± SEM ∗*p* < 0.05 or ∗∗*p* < 0.01, ∗∗∗ *p* < 0.001 *versus* WT controls (two-way ANOVA followed by Bonferroni corrected two-way Student’s *t* test).

To date, cathepsins B, L, C, and Z have all been implicated in inflammasome activation (17). Cathepsin Z was found to be sufficient for full IL-1β production after activation with nigericin, ATP, and MSU (8, 14). However, the mechanism of how cathepsin Z leads to increased IL-1β production remains elusive. To study the involvement of cathepsin Z in NLRP3-mediated IL-1β production, we derived bone marrow-derived dendritic cells (BMDC) and isolated peritoneal macrophages from WT and *Ctsz*^−/−^ mice and exposed them to silica crystals for 6 h. *Ctsz*^−/−^ APCs secreted significantly less IL-1β compared with WT APCs (Fig. 2). To determine whether cathepsin Z has a similar effect on IL-1β production in human APCs, we generated a *CTSZ*^−/−^ THP-1 monocytic cell line using the CRISPR-Cas9 system (Fig. S2*A*) (21). Indeed, compared with WT THP-1 cells, *CTSZ*^−/−^ THP-1 cells generated significantly lower levels of both total and bioactive IL-1β as determined by ELISA and the HEK-Blue bioactive IL-1β reporter system, respectively (Figs 2, *C* and *D* and S2*B*). Consistent with the findings in murine *Ctsz*^−/−^ APCs (8), IL-1β mRNA transcript levels were found to be equivalent between *CTSZ*^−/−^ and WT THP-1 cells as measured by qPCR, as were levels of caspase-1, NLRP3, and ASC transcripts (Fig. S2, *B*–*E*). Interestingly, we found lower levels of the 10 kDa subunit of caspase-1 in the supernatant by Western blot, suggesting that cathepsin Z also impacts the activation or secretion of caspase-1 (Fig. 3, *A*–*C*). The levels of active 17 kDa IL-1β were also lower in CTSZ^−/−^ THP-1 lysates (Fig. 3, *D*–*F*). This suggests a mechanism whereby cathepsin Z can impact IL-1β release through caspase-1 activation.

Cathepsin Z is specifically involved with the generation of IL-1β through the NLRP3 inflammasome as activation of the NLRC4 inflammasome using Flagellin, or the AIM2 inflammasome using dA:dT, did not result in differences in IL-1β generation by WT and *CTSZ*^−/−^ THP-1 cells (Fig. S3, *A* and *B*). Furthermore, no IL-1β secretion was observed without activation of the NLRP3 inflammasome *via* silica (Fig. S3*C*). To determine whether cathepsin Z is involved in pyroptosis or inflammatory cell death, we examined whether *CTSZ*^−/−^ THP-1 cells show altered rates of cell death by measuring lactate dehydrogenase release from the cell after NLRP3 inflammasome activation with silica. *CTSZ*^−/−^ THP-1 cells did not show a difference in cell death compared with WT THP-1 cells, which indicates that cathepsin Z was not involved in pyroptosis before IL-1β release (Fig. S4). These data suggest that cathepsin Z is involved in modulating the second signal of inflammasome activation, where caspase-1 is activated and cleaves IL-1β. Importantly, the absence of cathepsin Z did not result in a total ablation of IL-1β production but rather lowered the amount of secreted IL-1β (Fig. 2, *A*–*D*). Therefore, it is likely that cathepsin Z is a modifier in the activation of the NLRP3 inflammasome.

### The secreted proform of cathepsin Z is required for NLRP3-mediated IL-1β generation

**Figure 4:**
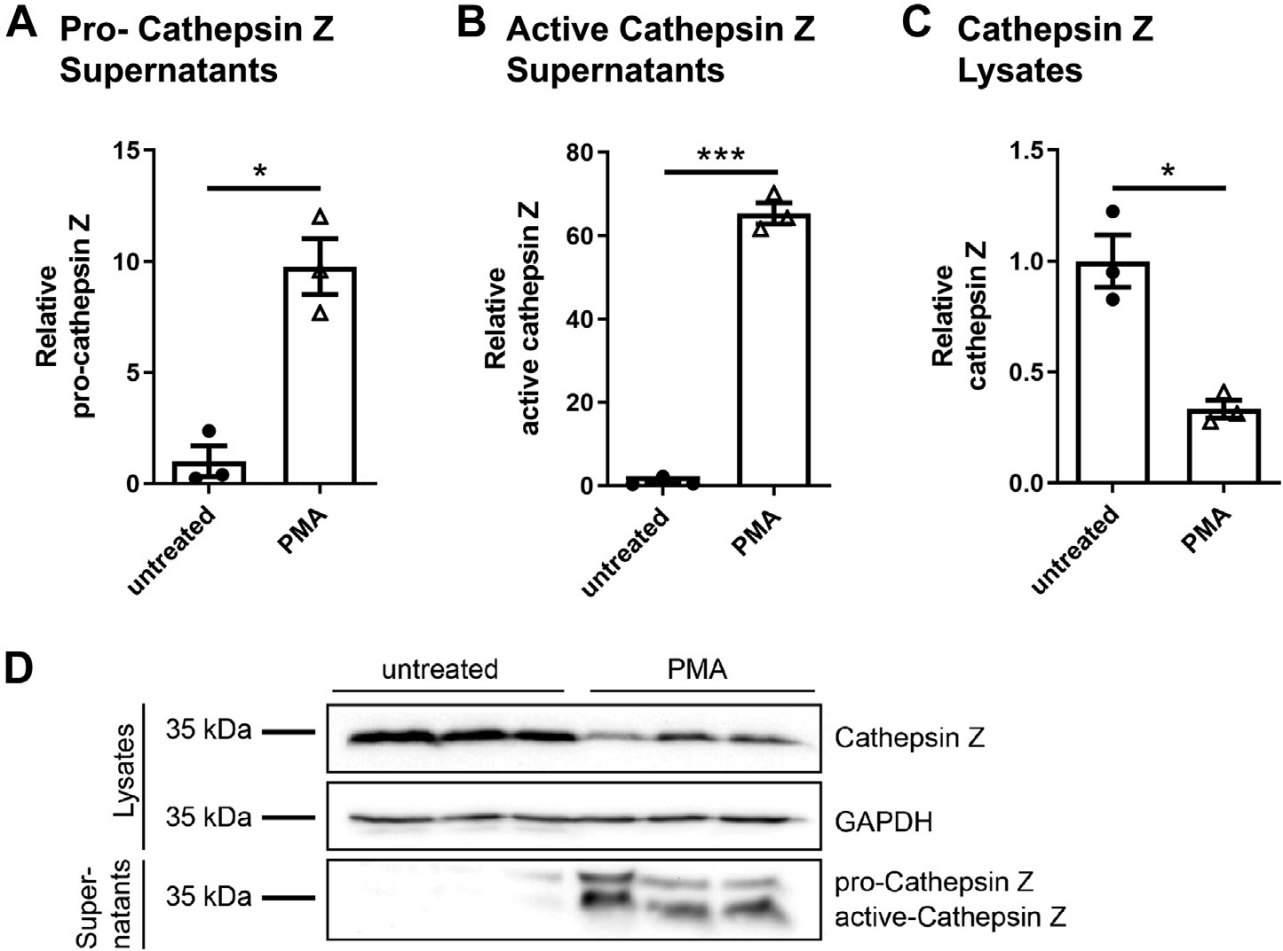
**Cathepsin Z is secreted into the extracellular space after differentiation of THP-1 cells usingphorbol myristate acetate (PMA).***A*–*D*, THP-1 cells were treated with 10 nM PMA overnight. *A*–*C*, Western blot quantification using densitometric analysis of total protein blots of secreted cathepsin Z (*A* and *B*) or cellular cathepsin Z (*C*) in THP-1 cells (n = 3). The data are presented as means ± SEM ∗*p* < 0.05 or ∗∗∗*p* < 0.001 *versus* WT controls (one-way ANOVA after Bonferroni corrected two-way Student’s *t* test). *D*, representative Western blot of cathepsin Z secreted from THP-1 cells treated with 10 nM PMA overnight.

**Figure 5:**
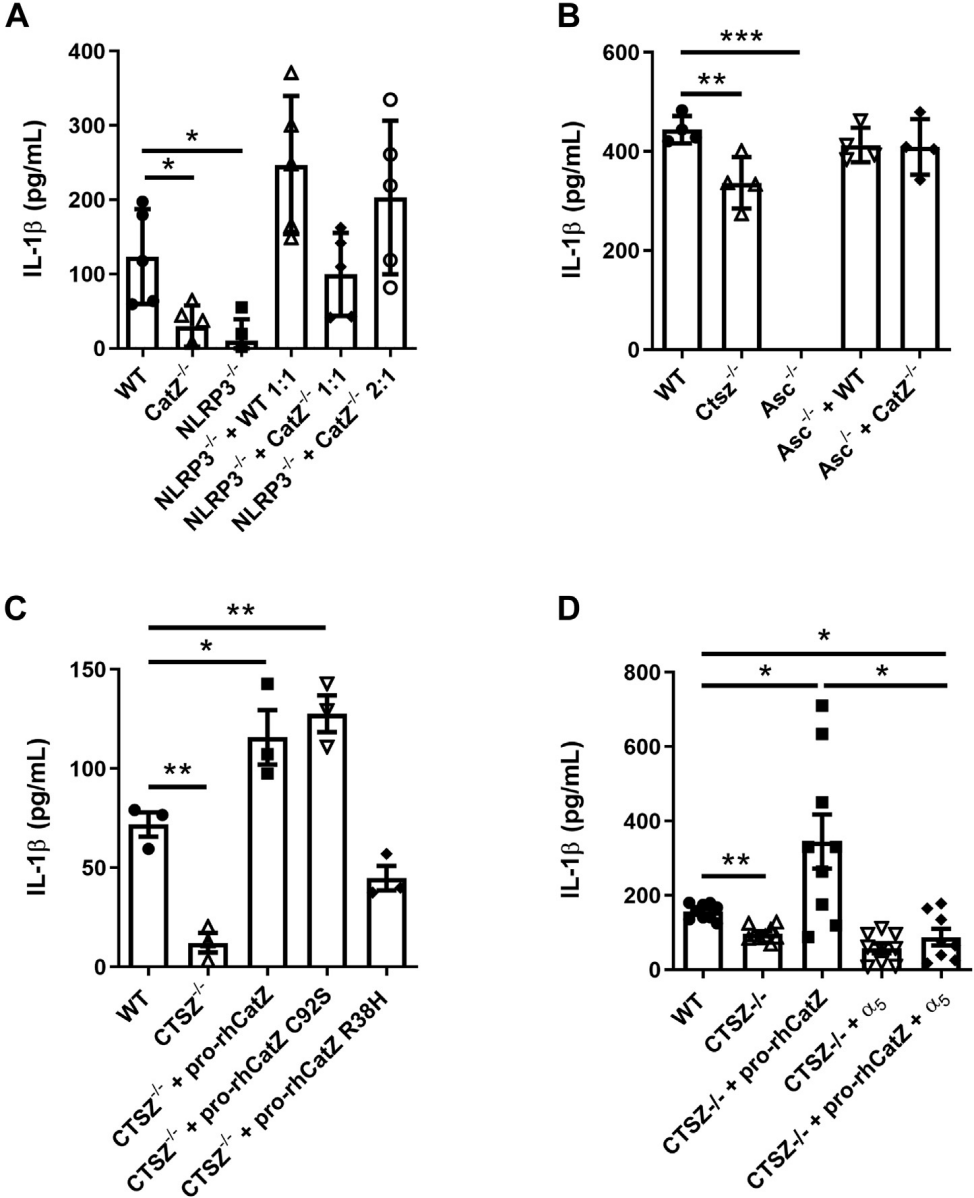
**Extracellular cathepsin Z can rescue the deficiency in secreted IL-1β from cathepsin Z deficient THP-1 and dendritic cells.***A*, WT, CTSZ^−/−^, or NLRP3^−/−^ THP-1 cells cultured alone or in combination with NLRP3^−/−^ THP-1 cells. IL-1β protein released from THP-1 cells measured by HEK-Blue assay (n = 5). *B*, WT, Ctsz^−/−^, or Asc^−/−^ DCs cultured alone or in combination with Asc^−/−^ DCs. IL-1β protein released from DCs measured by ELISA (n = 4). *C*, WT and CTSZ^−/−^ THP-1 cells were incubated with 10 nM phorbol myristate acetate (PMA) for 16 h with either 50 ng pro-rhCatZ, 50 ng pro-rhCatZ (C92S), or 50 ng pro-rhCatZ (R38H) after 128 μg silica for 6 h. *D*, WT and CTSZ^−/−^ THP-1 cells were incubated with 10 nM PMA for 16 h with either 50 ng pro-rhCatZ, 10 mg/ml α5-blocking antibody, or 50 ng pro-rhCatZ plus 10 mg/ml a5-blocking antibody after 128 μg of silica for 6 h. *C* and *D*, the levels of secreted IL-1β measured by HEK-Blue assay. The data are presented as means ± SEM ∗*p* < 0.05, ∗∗*p* < 0.01, or ∗∗∗*p* < 0.001 *versus* WT controls (one-way ANOVA followed by Dunnet’s test (n = 9)).

Extracellular cathepsin Z has been implicated in tumor metastasis and proliferation through its integrin-binding domain in a pancreatic tumor model (22). To explore the possibility of extracellular cathepsin Z influencing NLRP3 activation of APCs, we first set out to determine whether the lysosomal protease could be detected in THP-1 culture supernatants by Western blot. Indeed, both the pro and the active forms of cathepsin Z were secreted into the supernatant after THP-1 treatment with phorbol myristate acetate (PMA) for 16 h (Fig. 4, *A*–*D*). Correspondingly, the amount of cathepsin Z found in the cell lysates diminished with PMA treatment (Fig. 4*C*). A similar result was also observed in murine BMDC (Fig. S5). The secretion of cathepsin Z by activated THP-1 cells lends itself to the possibility that the protease may be functioning at an extracellular locale to contribute to NLRP3 inflammasome activation. To test this hypothesis, we cocultured *CTSZ*^−/−^ THP-1 cells with *NLRP3*^−/−^ THP-1 cells to provide extracellular cathepsin Z secreted from *NLRP3*^*−/−*^ THP-1 cells to the *CSTZ*^*−/−*^ THP-1 cells. (Fig. 5*A*). The *NLRP3*^−/−^ THP-1 cells do not produce IL-1β downstream of NLRP3 activation but would still secrete cathepsin Z after PMA treatment. The *CTSZ*^−/−^ THP-1 cells possess the machinery to produce an NLRP3 inflammasome mediated response, but they do not express cathepsin Z (and therefore do not secrete cathepsin Z into the extracellular space), leading to diminished generation of IL-1β in response to silica. Therefore, if extracellular cathepsin Z were contributing to NLRP3 inflammasome activation, the *NLRP3*^−/−^ THP-1 cells could supply extracellular cathepsin Z to restore the production of IL-1β from the *CTSZ*^−/−^ THP-1 cells. We found that when the *CTSZ*^−/−^ and *NLRP3*^−/−^ THP-1 cells were cultured together, IL-1β generation was partially restored (Fig. 5*A*). Similar restoration of IL-1β generation was found when *Ctsz*^−/−^ BMDC were cultured with BMDC from *ASC*^−/−^ mice (Fig. 5*B*). Coculture did not affect the rates of cell death (Fig. S6).

To more specifically determine the contribution of cathepsin Z to NLRP3 inflammasome activation, we generated WT and mutant versions of recombinant human cathepsin Z (rhCatZ), which was supplied to the *CTSZ*^−/−^ THP-1 cells before inflammasome activation with silica (Fig. 5*C*). This effect was specific to cathepsin Z, as cathepsin S was unable to rescue the CTSZ^−/−^ phenotype (Fig. S7). WT rhCatZ and the catalytically dead active site mutant C92S pro-rhCatZ were able to fully restore IL-1β production. In contrast, the RGD integrin-binding domain mutant R38H pro-rhCatZ protein was unable to rescue the IL-1β phenotype from the *CTSZ*^−/−^ THP-1 cells (Fig. 5*C*). Heat inactivated rhCatZ, pro-rhCatZ, or thrombin were unable to fully rescue IL-1β production (Fig. S8). Together, these data suggest that extracellular full-length cathepsin Z is required for the augmentation of NLRP3-generation of IL-1β and that this ability is dependent on the integrin-binding capacity of the enzyme, but independent of the proteolytic function of cathepsin Z.

### The ability of cathepsin Z to enhance NLRP3-generation of IL-1β is dependent on α_5_ integrin

It has been previously shown that a pathogen-derived enzyme that possesses an RGD integrin-binding domain (EhCP5, *Entamoeba histolytica*) enhanced the activation of the NLRP3 inflammasome and activation of macrophages through ligation of the α_5_β_1_ integrin (23). In support of the hypothesis that cathepsin Z was acting *via* the same mechanism, pro-rhCatZ was unable to amplify NLRP3-generation of the IL-1β *CTSZ*^−/−^ THP-1 cells after the blocking of α_5_ using a neutralizing antibody (Fig. 5*D*). This data demonstrates that cathepsin Z is secreted from THP-1 cells and can signal through the α_5_ integrin leading to modulation of the NLRP3 inflammasome through caspase-1 activation and IL-1β production from THP-1 cells.

## Discussion

Our data demonstrate the requirement of secreted cathepsin Z for enhanced production of IL-1β by APCs after NLRP3 inflammasome activation in response to silica. Furthermore, we have shown that full-length cathepsin Z is required to modulate the NLRP3 inflammasome. Ligation of the α_5_ integrin by the RGD integrin-binding domain, and not the catalytic activity, is the key determinant for this unexpected function of this lysosomal protease. This work further adds to the growing number of physiological and pathophysiological roles for cathepsin Z outside of its initially-attributed function as a lysosomal carboxypeptidase.

The integrin-binding function of extracellular cathepsin Z has also been described in the context of cancer. Akkari *et al.* (22) demonstrated that extracellular cathepsin Z can signal through integrins containing an RGD binding domain in a model of pancreatic cancer, leading to changes in FAK and Src phosphorylation. Cathepsin Z has also been shown to physically interact with the β_3_ integrin in non small cell lung cancer cells (24). An interesting example of integrin signaling and NLRP3 inflammasome activation comes from the amoeba *E. histolytica* where the cysteine protease EhCP5 can bind to both α_5_β_1_ and α_v_β_3_ integrins through an RGD sequence (23, 25). Engagement with α_v_β_3_ leads to activation of the PI3K–Akt pathway, NFκB activation, and upregulation of IL-1β (25), whereas activation of α_5_β_1_ results in the opening of pannexin 1, release of extracellular ATP, and NLRP3 inflammasome activation (23). The examples of integrin signaling leading to NLRP3 inflammasome activation are also found in bacteria. Td92 is a *Treponema denticola* protein that has integrin-binding function and can activate the NLRP3 inflammasome through α_5_β_1_ leading to ATP release (26). Although these examples come from the interaction of extracellular pathogens with human monocytes, they highlight the integrin-signaling pathway as another regulatory pathway that governs NLRP3 inflammasome activation. The interaction of cathepsin Z with the α_5_ integrin is the first example of an endogenous extracellular protein modulating the NLRP3 inflammasome through integrins. Whether or not cathepsin Z leads to the activation of downstream kinases including FAK, Src, and Akt remains to be investigated.

Unlike other cathepsins, cathepsin Z is unique in its ability to modulate NLRP3 inflammasome activation independently of other cathepsins, likely because of the unique integrin-binding site found in cathepsin Z (14). The use of CA-074-Me to inhibit cathepsins in NLRP3 inflammasome activation precludes cathepsin Z, as the active site of cathepsin Z is not required in this context (8, 14). Furthermore, the observation that the active site of cathepsin Z is not required in its modulatory capacity of the NLRP3 inflammasome suggests the functional requirement of pH 5.0 for cathepsin Z protease activity is not an issue in the extracellular environment (27). Nevertheless, it is noteworthy that by Western blot, we were able to detect both the pro and active forms of cathepsin Z secreted from THP-1 cells. Cathepsin Z has been found secreted from human osteoblasts, where it is active and digests CXCL-12 (28). Therefore, there remains the possibility of a role for active cathepsin Z secreted from macrophages during inflammation, outside of the context of NLRP3 inflammasome activation.

Given the observation that the absence of cathepsin Z does not completely prevent inflammation from occurring in our silicosis model, it is possible that there are NLRP3-independent processes contributing to the development of silicosis, as has previously been reported (7, 29). Leukotriene B_4_ has been identified as an important mediator of lung inflammation after silica deposition, independent of NLRP3 inflammasome activation (29). It is also possible that cathepsin Z has another function in this inflammatory model. Active cysteine cathepsins have been detected in the bronchoalveolar lavage fluid from patients suffering from silicosis, suggesting the importance of active cysteine proteases in the development of silicosis, potentially in tissue remodeling (30). To support this, we observed slightly lower levels of fibrosis in the *Ctsz*^−/−^ animals. Therefore, it is possible that cathepsin Z’s contribution to inflammation in the mouse model of silicosis may involve both IL-1β generation and other inflammatory processes.

Previous work from our lab outlined the role for cathepsin Z in IL-1β production in EAE (8). Huynh *et al.* (9) reported the hypomethylation of the *CTSZ* locus in pathology-free regions of MS brains, which corresponded with an increase in *CTSZ* mRNA. *Ctsz* mRNA is also increased in the central nervous system of mice with EAE (8, 31). The development of MS involves a complex interplay of the innate and adaptive immune systems, including the NLRP3 inflammasome and IL-1β production (32). IL-1β appears to be a critical mediator of EAE, as IL-1β and NLRP3 inflammasome component knockout models lead to a reduction in EAE pathology (8, 9, 32). The finding that extracellular cathepsin Z can signal through the α_5_β_1_ integrin to enhance IL-1β production has important implications in MS and supports the possibility that cathepsin Z found in the brain may be secreted from resident microglia and may have an important function modulating inflammation in the brain (33, 34).

Further to this concept, cathepsin Z has been proposed as a biomarker of inflammation in a number of different models due to its secretion into the urine, detection of mRNA in blood mononuclear cells, and protein detection in the blood (35, 36, 37, 38). To date, cathepsin Z has been found to be involved in cancer models (22, 39, 40), rheumatoid arthritis (41), Alzheimer’s disease (42), Sjogren’s syndrome (43), and primary biliary cholangitis (44). This range of diseases linked to cathepsin Z suggests that cathepsin Z may be characterized as an important biomarker of inflammation.

This work adds to the growing body of evidence that the nonproteolytic function of cathepsin Z is as least as important as its protease function in specific contexts (22, 45). Although we have identified a target for cathepsin Z, there is still much to learn regarding the downstream implications of cathepsin Z ligation of the α_5_ integrin leading to NLRP3 inflammasome activation and IL-1β production. Further work should focus on how extracellular signaling leads to modulation of the NLRP3 inflammasome and the role of integrin signaling in NLRP3 inflammasome activation. This work highlights yet another mechanism of NLRP3 inflammasome regulation and emphasizes the complex regulatory networks involved in NLRP3 inflammasome activation.

## Experimental procedures

### Mice and cells

All animal research was performed in accordance with the Canadian Council for Animal Care, and protocols were approved by the University of Calgary Animal Care and Use Committee. All mice were used at 6 to 8 weeks of age. The female mice were used for all silicosis experiments, and the male and female mice were used for isolation of BMDC. C57BL/6 (WT) mice were purchased from the Jackson Laboratory. *Ctsz*^*−/−*^ mice were generated and generously gifted by Thomas Reinheckel (Albert-Ludwigs-University) (40). Asc-deficient mice (*Pycard*^−/−^) were generously gifted by Dan Muruve (University of Calgary) (46). BMDC were derived from C57BL/6 and *Ctsz*^−/−^ murine bone marrow, as previously described (8, 47). All the cells were cultured in tissue culture-treated plates and grown in a 5% CO_2_ 37 °C incubator. In brief, bone marrow was isolated and cultured in dendritic cell media (20% supernatant from J558L myeloma cell line [CVCL_3949] transfected with GM-CSF cDNA, 10% v/v fetal bovin serum (FBS), 0.5 mM β-mercaptoethanol, 50 U/ml penicillin streptomycin, 2 mM L-glutamine, and 10 mM Hepes in RPMI) to allow for differentiation (8). The *NLRP3*^*−/−*^ THP-1 cells generated by CRISPR knockout, as previously described (48). THP-1 cells (ATCC TIB-202) were grown in suspension in THP-1 media (RPMI supplemented with 10% v/v FBS, 10 mM Hepes, 50 U/ml penicillin-streptomycin, and 0.5 mM β-mercaptoethanol). Peritoneal macrophages were isolated from the peritoneal cavity in 8 ml of 1× PBS using an 18 gauge needle. The cavity was massaged gently before the removal of 6 ml of PBS/peritoneal fluid. The cells were washed in RPMI with 10% FBS, and plated on tissue culture treated plates (49).

### Generation of CRISPR *CTSZ* knockout THP-1 cells

gRNA for cathepsin Z was generated using the Zhang lab CRISPR design guide (crispr.mit.edu) and cloned into the lentiCRISPRv2 plasmid (CTACTTCCGCCGGGGACAGACC) (21, 50). Two additional guides were designed and tested. LentiCRISPRv2 puro was a gift from Brett Stringer (Addgene plasmid # 98290; http://n2t.net/addgene:98290; RRID:Addgene_98290). The cloning was performed according to Sanjana *et al.* 2014 (21). Lentivirus was produced by transfecting the cathepsin Z targeted-gRNA lentiCRISPRv2 construct with the psPAX2 and pCMV-VSVG plasmids into HEK293T cells. psPAX2 was a gift from Didier Trono (Addgene plasmid # 12260; http://n2t.net/addgene:12260; RRID:Addgene_12260) (51). pCMV-VSV-G was a gift from Bob Weinberg (Addgene plasmid # 8454; http://n2t.net/addgene:8454; RRID:Addgene_8454). The virus was harvested from the supernatant and concentrated at 122,000*g* for 2 h. The concentrated virus was applied to THP-1 cells, and the transduced cells were selected using puromycin. The surviving cells were plated in limiting dilutions to produce monoclonal cell lines, which were subsequently evaluated for the expression of cathepsin Z.

### Western blotting

The cells were lysed in RIPA buffer (150 mM NaCl, 25 mM sucrose, 50 mM Tris pH8.0, 1 mM EDTA pH 8.2, 1% v/v Triton-X, 0.1% w/v SDS, Protease Inhibitor Cocktail Set I [Calbiochem] as per manufacturer’s instructions, and 0.5 mM DTT) for 10 min on ice. The samples were snap frozen, thawed, and sample buffer was added. The supernatants were frozen at −80 °C with 8.5% w/v TCA and 0.1% w/v sodium lauroyl sarcosinate (52), thawed, and centrifuged at 10,000*g* at 4 °C for 10 min. The pellet was suspended in acetone to wash and centrifuged at 10,000*g* at 4 °C for 10 min. The resulting pellet was air dried and resuspended in RIPA buffer after the addition of sample buffer. Relative protein concentration was calculated using the Nanophotometer N60/N50 (Implen). The samples quantified by densitometry were run on Mini-PROTEAN TGX Stain-Free gels (BioRad). Cathepsin Z antibody (abcam; ab182623) and GAPDH antibody (Cell Signaling Technology; 2118S [14C10]) were used at 1:1000. Caspase-1 antibody (Santa Cruz; sc622 [C2315]) was used at 1:250 and active IL-1β at 1:500 (Cell Signaling, 83186S [D3A3Z]). Pro-IL-1β used at 1:1000 (Cell Signaling Technology; 12242S [Lot 1]). Integrin alpha-5 antibody (Santa Cruz; sc10729 [H-104]) and Cas9 (Cell Signaling Technology; 14697T [7A7-3A3]) were used at 1:1000. The samples blotted with cathepsin Z, alpha-5, cas9, and GAPDH antibodies were imaged using the ECL substrate Immobilon Crescendo Western HRP Substrate (MilliporeSigma). The samples blotted with caspase-1 antibody were imaged using the high sensitivity ECL substrate SuperSignal West Femto Maximum Sensitivity Substrate (Thermo Fisher Scientific). Densitometry was calculated using ImageLab software (BioRad).

### Quantitative PCR

RNA from THP-1 cells and lung tissue was isolated using the RNeasy Mini Kit (Qiagen), according to manufacturer’s instructions. cDNA was reverse-transcribed using the qScript cDNA super kit (QuantaBio). qPCR was run using the SYBR Green Master Mix (BioRad) and the iQ5 Real-Time PCR System (BioRad). Human primers are as follows: cathepsin Z forward (TGTCATTGACTGTGGCAATGCTGG), cathepsin Z reverse (TGCAGGTCCCACACTGGTTAAACT); IL-1β forward (ACAGATGAAGTGCTCCTTCCA), IL-1β reverse (GTCGGAGATTCGTAGCTGGAT); ASC forward (AGTTTCACACCAGCCTGGAA), ASC reverse (TTTTCAAGCTGGCTTTTCGT); caspase-1 forward (GAAGGACAAACCGAAGGTGA), caspase-1 reverse (GGTGTGGAAGAGCAGAAAGC); and NLRP3 forward (CTTCTCTGATGAGGCCCAAG), NLRP3 reverse (GCAGCAAACTGGAAAGGAAG). Mouse primers are as follows: cathepsin Z forward (CCTGTCCGGGAGGGAGAA), cathepsin Z reverse (CTGGTCCGGCAATAGTTGGT); IL-1β forward (CAACCAACAAGTGATATTCTCCATG), IL-1β reverse (ACGTCGACCTCTCACACCTAG); and caspase-1 forward (ACTGGGACCCTCAAGTTTTG), caspase-1 reverse (CAACCCTCGGAGAAAGATGT).

### Recombinant cathepsin Z

Recombinant human cathepsin Z was epitopically tagged with FLAG (DYKDDDDK) and 6xHis6, cloned into the pcDNA3.1(+) mammalian expression vector (V79020, Thermo Fisher Scientific), and expressed in HEK293 cells (ATCC CRL-1573). In brief, human cathepsin Z was amplified by PCR with *Hin*dIII (5′) and *Bam*HI (3′) flanking restriction enzyme cut sites from the pTRIPz plasmid (a kind gift from Dr Thomas Reinheckel, Albert-Ludwigs-Universität Freiburg) (forward primer: ACACACAAGCTTCCACCATGGCGAGGCGCGGGCCAG and reverse primer: ACACACGGATCCCGAACGATGGGGTCCCCAAATG). The PCR product was cloned *via* the pCR2.1-TOPO shuttle vector into pCDNA3.1(+) (Invitrogen) using *Hin*dIII and *Bam*HI (New England Biolabs). The plasmid was carried in a-select *Escherichia coli* (BIO-85027, Bioline), and the DNA for screening was isolated using the E.Z.N.A. Plasmid Mini Kit I (D2500-02, Omega Biotek). The positive clones were identified by PCR and confirmed by sequencing by Eurofins Genomics.

FLAG and hexahistidine epitope tags were added by primer insertion using T4 DNA ligase after restriction digest with *Bam*HI, (forward primer: GATCCAGGAGGAGACTACAAAGACGATGACGACAAGCACCACCACCACCACCACTAAG and reverse primer: GATCCTTAGTGGTGGTGGTGGTGGTGCTTGTCGTCATCGTCTTTGTAGTCTCCTCCTG). Positive clones were identified by sequencing.

The LPKN cathepsin L cut site found in human cathepsin Z was substituted with a “LVPRGS” thrombin recognition site by site-directed mutagenesis using the Q5 Site-Directed Mutagenesis kit following the manufacturer’s instructions (New England Biolabs) (forward primer: CGCGGGGATCCTGGGACTGGCGCAATGT and reverse primer: GTACCAGATCTGCTGGGGACAGGTACTCATG). Finally, the integrin-binding domain and the active site of cathepsin Z were mutated using the same strategy. The arginine of the integrin-binding domain (R38) was substituted for a histidine, and the active site cysteine (C92) was substituted for a serine (R38H forward primer: CGGGCTAGCTCCGCTGGGGCGCAGC and reverse primer: TCCCCGTGCAGAGGCCGGTAGCAGGTCTG; C92S forward primer: AATACTGCGGATCCTCCTGGGCCCAC and reverse primer: GGGGGATGTGCTGGTTCC). The positive clones were screened by PCR and confirmed by sequencing. Plasmids used for HEK293 cell transfection with Lipofectamine 3000 (Invitrogen) were isolated using the PureYield Plasmid Midiprep System (Promega).

The cells stably expressing cathepsin Z were selected using G418 and screened for expression of WT recombinant human cathepsin Z (WT-rhCatZ), R38H mutated rhCatZ (R38H-rhCatZ), or C92S mutated rhCatZ (C92S-rhCatZ). The recombinant cathepsin Z was isolated after the lysis of cells with 300 mM NaCl and 50 mM NaPO_4_ (pH 7.4), and purified using His60 Ni Superflow Resin (Clontech Laboratories Inc). Activated cathepsin Z was produced by incubating beads with 10 U of human thrombin (Sigma-Aldrich) for 30 min at 37 °C in 20 mM Tris-HCl and 100 mM NaCl (pH 8.0). The protein was eluted using 300 mM NaCl, 50 mM NaPO4, 600 mM imidazole (pH 7.4), and dialyzed overnight. The activity of the isolated protein was determined using Z-Phe-Arg-AMC (AnaSpec Inc). WT and mutant recombinant protein (50 ng, as determined by Bradford assay) was applied to each well of a 364-well plate in 200 mM sodium citrate (pH 5.0), 0.2 M NaCl, 1 mM EDTA, and 500 μM cysteine:cystine (500:1 M ratio) and 5 μM Z-Phe-Arg-AMC added immediately before the plate read using the FLUOstar OPTIMA microplate reader (PerkinElmer Life Sciences). The liberated fluorescence was made relative to background over 30-min. For cell-based assays, 50 ng of recombinant protein was applied to each well of a 96-well plate.

### Murine model of silicosis

After general anesthesia with ketamine-xylazine (Narketan, Vetoquinol), 10 mg of MIN-U-SIL Silica (U.S. Silica Holdings Inc) suspended in 40 μl PBS or PBS alone were introduced into airways through the oropharyngeal aspiration method, as previously described (53). In brief, 40 μl of liquid containing suspended silica was placed on the distal portion of the tongue while simultaneously pinching the nares and holding the tongue out of the mouth. After recovery from anesthesia, the mice were housed for 90 days under routine husbandry conditions with daily monitoring. On day 90-post aspiration, the mice were euthanized and lungs removed. The right lung lobe was snap frozen in liquid nitrogen for RNA isolation. The right bronchi were closed using haemostats, and the remaining lungs were infused with 10% neutral buffered formalin. Standardized images were taken for gross pathology. Standardized sections of the lungs were stained with H&E, and Massons’ trichrome (Prairie Diagnostic Services). The lungs were imaged for silica content, and the percentage of silica within each lobe was calculated using Fiji (54, 55).

Morphometric Analysis was performed on lung sections within slides stained with H&E. An ACVP board-certified veterinary pathologist assessed the extent of inflammation within silicosis lungs, grading each lung for the percent tissue affected, the number of foci, and the severity of inflammation for each lung lobe isolated from WT, *Ctsz*^*−/−*^ from control and silicosis groups. These values were summed to give a combined severity score and compared with the percent silica in each lung. The percent tissue affected was graded on a scale of 0 to 5 where 0 – no inflammation, 1 – 1 to 5% tissue affected, 2 – 6 to 10% tissue affected, 3 – 11 to 15% tissue affected, 4 – 16 to 20% tissue affected, 5 – 21 to 25% tissue affected. The number of inflammatory foci in all lung lobes was graded on a scale of 0 to 7 where 0 – no foci, 1 – 1 to 20 foci, 2 – 21 to 40 foci, 3 – 41 to 60 foci, 4 – 61 to 80 foci, 5 – 81 to 100 foci, 6 – 101 to 120 foci, and 7 – 121 to 140 foci. Inflammation severity was graded on a scale of 0 to 4, where 0 – no inflammation, 1 – minimal inflammation, low numbers of inflammatory cells in the bronchial lumen and peribronchial (<20 in total), 2 – mild inflammation, immediately peribronchial infiltration of inflammatory cells (<10 cells thick), 3 – moderate inflammation, inflammatory cells filling into the lumen of bronchi and peribronchial infiltrations of inflammatory cells that are 10 to 50 cells thick, and 4 – severe inflammation, peribronchial infiltrations that extend into the surrounding alveolar parenchyma and bridge between inflammatory foci. The combined severity score was calculated by summing the % tissue affected, the number of foci, and the severity score. Degree of fibrosis was graded on a scale of 0 to 3 where 1 – no fibrosis noted, 1 – mild fibrosis, approximately equal proportions of neutrophils and macrophages, 2 – moderate fibrosis, higher percentage of neutrophils to macrophages, and 3 – severe fibrosis, higher percentage of macrophages to neutrophils. The representative images were selected based on equivalent levels of silica found in each lobe between genotypes.

### NLRP3 inflammasome activation assays

THP-1 cells were treated with 10 nM of PMA for 16 h in a 96-well plate after 640 μg/ml of silica for 6 h. Recombinant cathepsin Z or cathepsin S (C402-10 μg, Novoprotein) were applied at 50 ng per well with silica. Recombinant cathepsin Z was heat inactivated for 10 min at 95 °C. For NLCR4 activation of THP-1 cells, the cells were treated with 10 nM PMA for 16 h after 2 mg/ml recombinant flagellin protein (NBP2-35882 [Lot D66102111]; Novus Biologicals) for 6 h. The experimental replicates (“n”) for THP-1 and are defined as cells grown in separate T75 flasks before plating. Dendritic cells were treated with 200 ng of LPS for 16 h after 640 μg/ml of silica for 6 h or 5 mM ATP for 30 min. For AIM2 activation of BMDC, the cells were treated with 100 ng LPS for 2 h after 2.5 mg/ml dA:dT (tlrl-patn; Invivogen) transfected with Lipofectamine 3000 (L3000001, Thermo Fisher Scientific) according to manufacturer’s instructions for 6 h. The experimental replicates for BMDC experiments are defined as an individual mouse. The supernatants from cells were removed and frozen at −80 °C for further use. Where indicated, 10 μg/ml of the antibody α_5_ (sc-10729, Santa Cruz) was applied to each well of a 96-well plate with or without recombinant protein for blocking assays.

### ELISA

After the activation of BMDC or THP-1 cells as described above, the supernatants were collected, and the concentrations of secreted IL-1β were measured using the OptEIA IL-1β ELISA kit (Becton Dickinson) or the mouse IL-1β ELISA Ready-SET-Go! (88-7013-88; eBioscience) for human or mouse samples, respectively, according to the manufacturer’s instructions. The resulting signal was measured using a 2104 EnVision Multilabel Plate Reader (PerkinElmer).

### Bioactive IL-1β detection assay

After the activation of THP-1 cells, as described above, the supernatants were collected, and the concentration of secreted IL-1β was measured using the HEK-Blue IL-1β reporter cells and QUANTI-Blue secreted alkaline phosphatase detection medium (Invivogen). The resulting signal was measured using a 2104 EnVision Multilabel Plate Reader.

### Cell death detection assay

After the activation of THP-1 cells, as described above, the supernatants were collected, and the levels of LDH were measured with the Pierce LDH Cytotoxicity Assay Kit (Thermo Fisher Scientific), according to manufacturer’s instructions. The resulting signal was measured using a 2104 EnVision Multilabel Plate Reader. The levels of LDH released in PMA and silica-treated THP-1 cells was compared with THP-1 cells treated with the positive control provided.

### THP-1 co-culture assays

The NLRP3^−/−^ THP-1 cells were cultured with WT or CTSZ^−/−^ at a ratio of 1:1 or 1:2. The cells were activated with 10 nM PMA for 16 h after 128 μg silica for 6 h. The supernatants were collected, and the concentration of secreted IL-1β was measured using HEK-Blue IL-1β reporter cells.

### Statistical analysis

Statistical analyses were performed by one-way ANOVA with a Dunnett’s post hoc test, by two-way ANOVA with Bonferroni-corrected unpaired two-way Student’s *t* test or by Bonferroni-corrected unpaired Student’s two-way *t* test, as specified (∗*p* < 0.05, ∗∗*p* < 0.01, ∗∗∗*p* < 0.001). The analysis of silicosis histology scoring was performed using the nonparametric Mann-Whitney U test. The analyses were completed using GraphPad Prism 8 (GraphPad Software).

## Data availability

All data presented is contained within this article.

## Supporting information

This article contains supporting information.

## Conflict of interest

The authors declare that they have no conflicts of interest with the contents of this article.
