# Supplementary material for: Extracellular cathepsin Z signals through the α5 integrin and augments NLRP3 inflammasome activation
Source: J Biol Chem. 2021 Dec 2;298(1):101459. doi: 10.1016/j.jbc.2021.101459 (PMC8753182; doi:10.1016/j.jbc.2021.101459)
Supplement: Supplemental Figures S1–S8 [file mmc1.pdf]

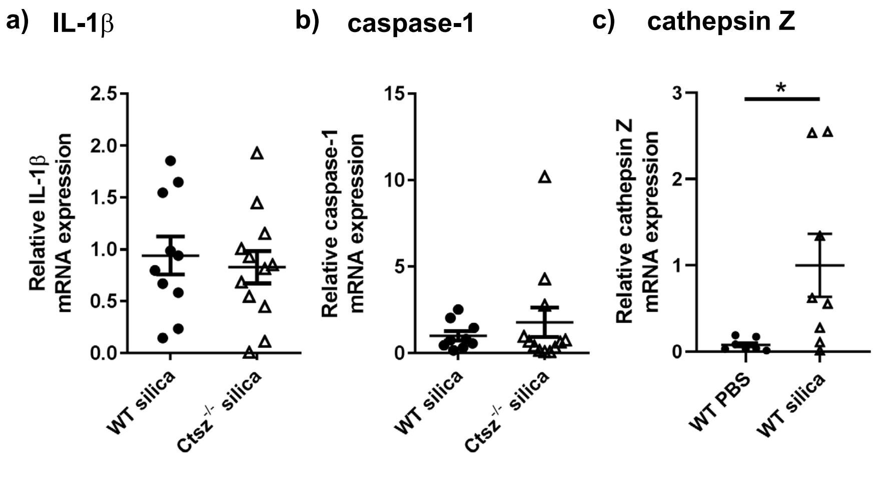

Supplemental Figure 1. **Caspase-1 and IL-1β mRNA expression is not impacted by knockout of cathepsin Z in silicosis.** Mice were treated by oropharyngeal aspiration of 10 mg silica. Samples were taken at 30 days post silica aspiration. **a-c)** mRNA transcript levels from silica and vehicle control (PBS) samples (n=10-13). **a)** IL-1β, **b)** caspase-1, and **c)** cathepsin Z mRNA. Data are presented as means ± SEM *$p < 0.05$ versus WT controls (two-way Student's t-test).

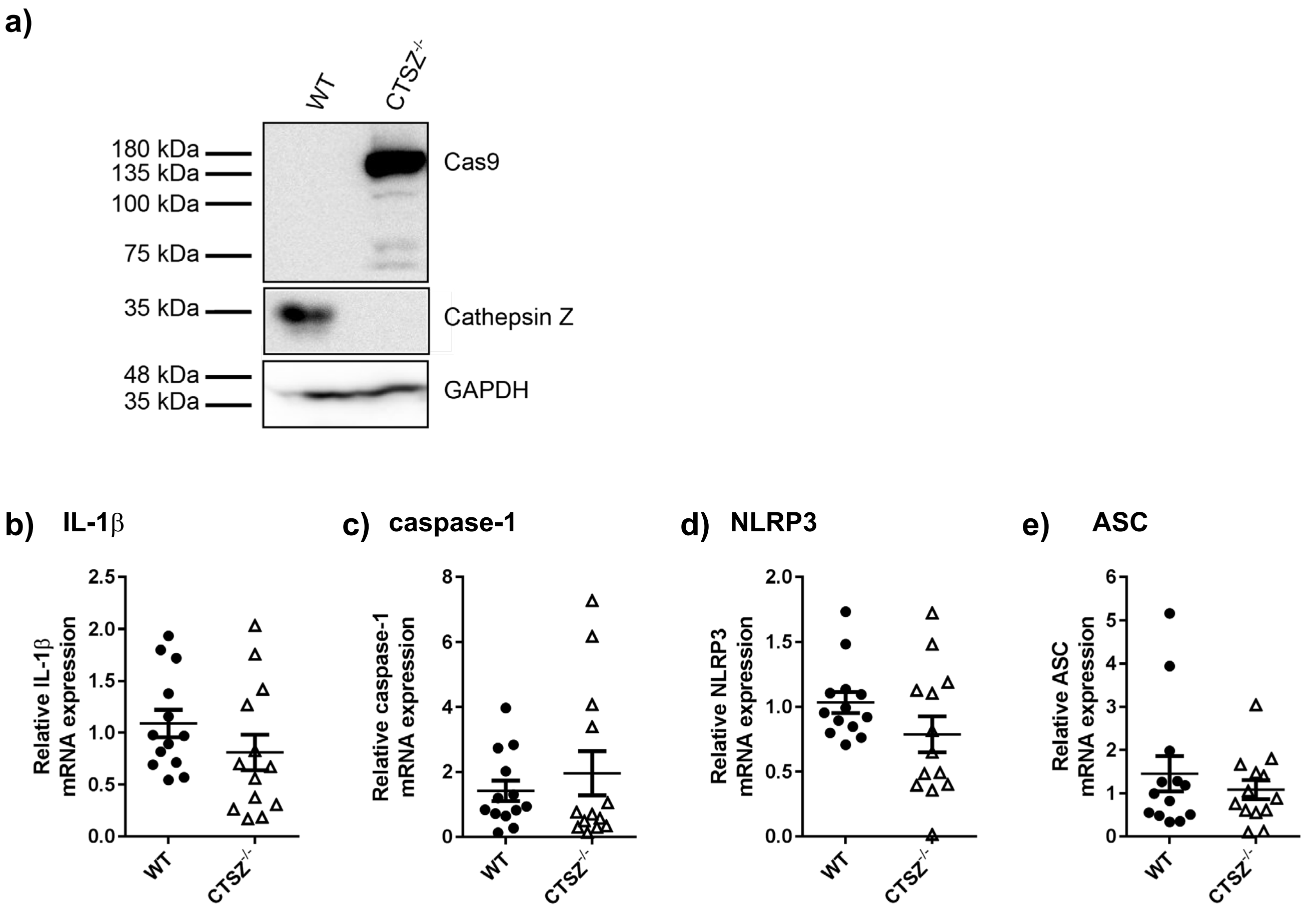

Supplemental Figure 2. **Cathepsin Z-/- THP-1 cells do not show differences in the transcription of inflammasome components. a)** Western blot of WT and CTSZ$^{-/-}$, Cas9-expressing THP-1 lysates. THP-1 cells treated with 10 nM PMA for 16 hours. **b-e)** mRNA transcript levels of **b)** IL-1β, **c)** caspase-1, **d)** NLRP3 and **e)** ASC from WT and CTSZ$^{-/-}$ THP-1 measured by RT-qPCR. Data are presented as means ± SEM **p < 0.01 versus WT controls (two-way Student's t-test (n=6-13)).

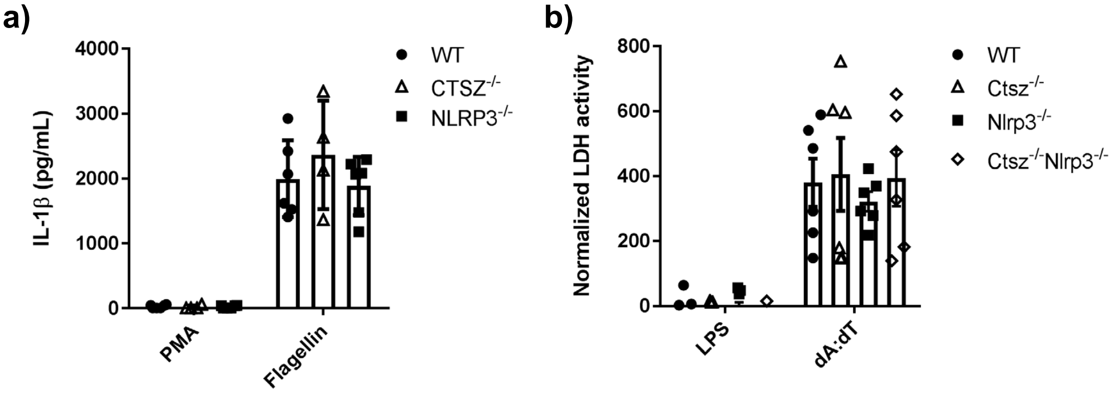

Supplemental Figure 3. **The presence of cathepsin Z does not influence NLRC4 or AIM2 inflammasomes. a)** WT, CTSZ$^{-/-}$ and NLRP3$^{-/-}$ THP-1 cells were activated with 10 nM PMA for 16 hours followed by 2 mg/mL flagellin for 6 hours. IL-1β protein levels were measured by ELISA (n=4-6). **b)** WT, Ctsz$^{-/-}$, Nlrp3$^{-/-}$, Ctsz$^{-/-}$Nlrp3$^{-/-}$ BMDC were activated with 100 ng LPS for 2 hours followed by 2.5 mg/mL dA:dT for 6 hours. Data are presented as means ± SEM versus WT controls (two-way ANOVA followed by Dunnett's test (n=5)).

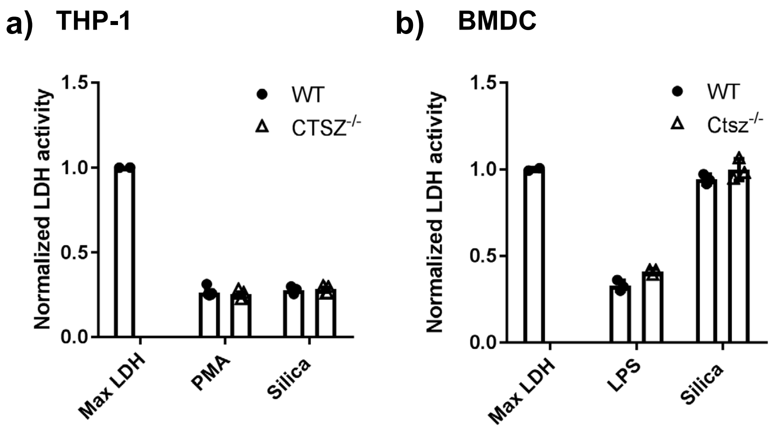

Supplemental Figure 4. **LDH release from WT and CTSZ$^{-/-}$ antigen presenting cells following activation indicates equal levels of cell death.** LDH release measured in supernatants following activation of **a)** THP-1 cells and **b)** BMDC with **a)** 10nM PMA for 16 hours, 128 µg silica for 6 hours or **b)** 100ng LPS for 2 hours, 128µg silica for 6 hours. Data are presented as means ± SEM versus WT controls (one-way ANOVA (n=3)).

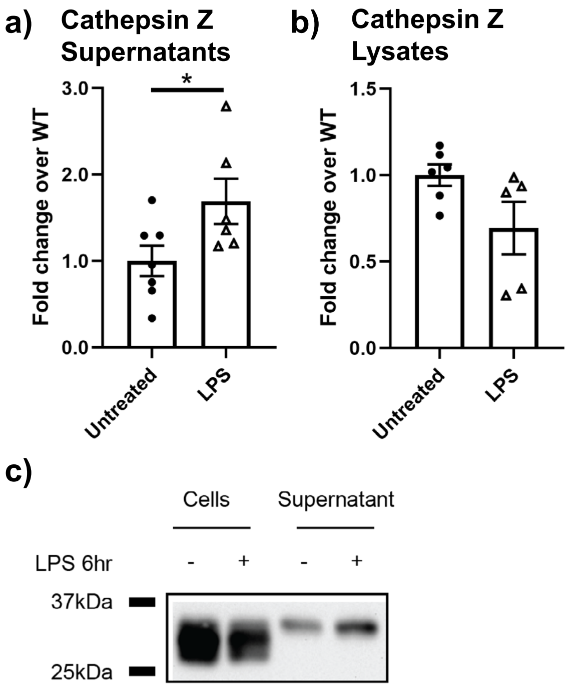

***Supplemental Figure 5.* Cathepsin Z is secreted into the extracellular space following activation of BMDC with LPS. a-c)** BMDC were either untreated or treated with 200 ng LPS for 6 hours. **a, b)** Western blot quantification using densitometric analysis of total protein blots of secreted cathepsin Z **(a)** or cellular cathepsin Z **(b)** in BMDC (n=5-6). Data are presented as means ± SEM *p < 0.05 versus WT controls (two-way Student's t-test). c) Representative western blot of cathepsin Z secreted from BMDC following treatment with LPS.

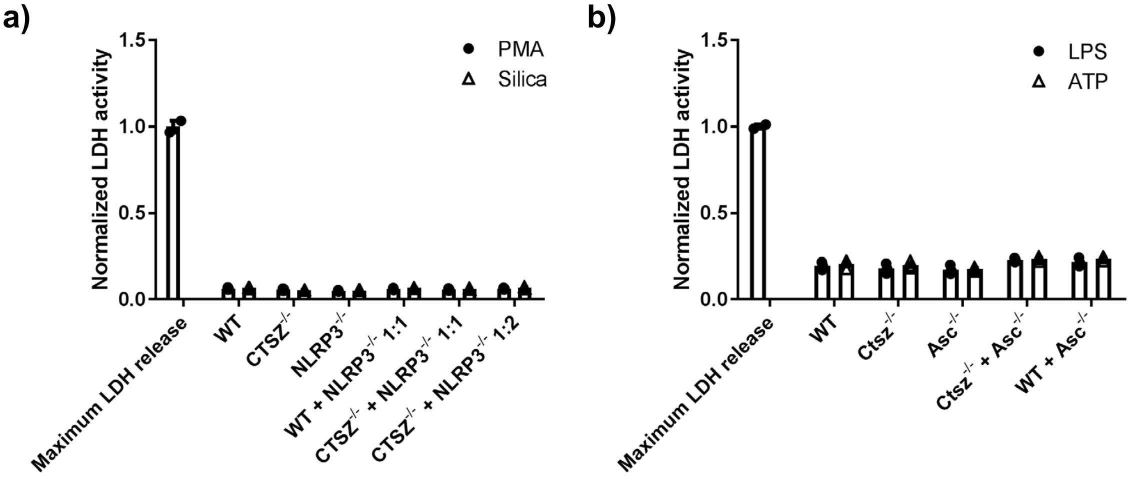

Supplemental Figure 6. **Co-culture of CTSZ$^{-/-}$ THP-1 and Ctsz$^{-/-}$ BMDC with WT and NLRP3$^{-/-}$ or Asc$^{-/-}$ cells does not impact cell death.** LDH release from THP-1 cells **a)** or BMDC **b)** measured following treatment with **a)** 10 nM PMA for 16 hours followed by 128 µg silica for 6 hours or **b)** 100 ng LPS for 2 hours followed by 5 mM ATP for 30 minutes. Data are presented as means ± SEM versus WT controls (one-way ANOVA followed by Bonferroni corrected two-way t-tests (n=4-6)).

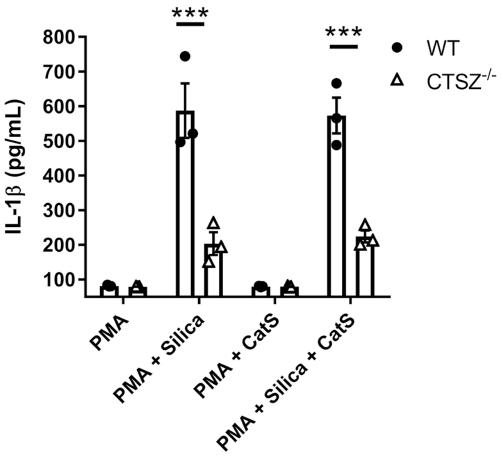

Supplemental Figure 7. **Addition of recombinant cathepsin S to CTSZ$^{-/-}$ THP-1 cells does not rescue IL-1β production.** WT and CTSZ$^{-/-}$ THP-1 cells were incubated with 10 nM PMA for 16 hours with 50 ng recombinant cathepsin S followed by 128 μg silica for 6 hours. Levels of secreted IL-1β measured by HEK-Blue assay. Data are presented as means ± SEM ***$p < 0.001$ versus WT controls (two-way ANOVA followed by two-way Student's t-test (n=3)).

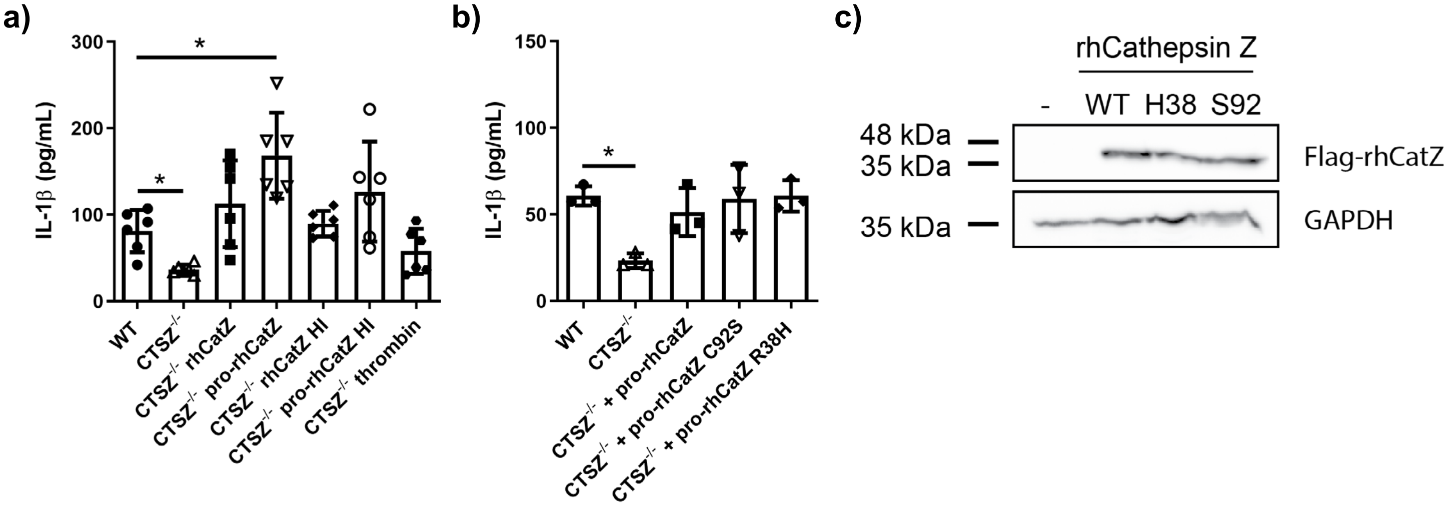

Supplemental Figure 8. **Recombinant cathepsin Z can rescue IL-1β generation from CTSZ$^{-/-}$ THP-1 cells. a, b)** Recombinant human cathepsin Z was isolated from HEK293 cells. Recombinant cathepsin Z was cleaved using thrombin. IL-1β was measured by HEK-Blue assay. **a)** 50 ng of human cathepsin Z cleaved (rhCatZ) or in pro-form (pro-rhCatZ) was incubated with CTSZ-/- THP-1 cells for 16 hours with 10 nM PMA. **b)** WT cleaved cathepsin Z (rhCatZ), active site mutant cathepsin Z (rhCatZ C92S) or integrin-binding site mutant cathepsin Z (rhCatZ R38H) were incubated with CTSZ$^{-/-}$ THP-1 cells for 16 hours with 10 nM PMA. Data are presented as means ± SEM versus WT controls *$p < 0.05$, **$p < 0.01$ (one-way ANOVA followed by Dunnett's test (n=6)). **c)** Western blot of HEK293 cells alone (-) or expressing FLAG-tagged rhCatZ WT (WT), R38H mutant (H38), or C92S (S92) mutant. Blots were probed with anti-FLAG.
